# Trends in Acute Pancreatitis-Related Mortality Among US Adults from 1999 to 2020

**DOI:** 10.1016/j.gastha.2025.100615

**Published:** 2025-01-10

**Authors:** Chun-Han Lo, Rahul Pannala, N. Jewel Samadder

**Affiliations:** 1 Department of Internal Medicine, Kirk Kerkorian School of Medicine at UNLV, Las Vegas, Nevada; 2 Division of Gastroenterology and Hepatology, Department of Medicine, Mayo Clinic, Phoenix, Arizona; 3 Department of Clinical Genomics, Mayo Clinic, Phoenix, Arizona; 4 Center for Individualized Medicine, Mayo Clinic, Phoenix, Arizona

Acute pancreatitis imposes a significant burden on the US health-care system, resulting in approximately 300,000 hospital admissions annually and generating costs exceeding $2 billion.^1^ Although mortality rates for acute pancreatitis have consistently declined over the past 2 decades,^2^ there is a lack of systematic analyses and robust estimates of these trends. In this cross-sectional study, we presented the age-adjusted mortality rates (AAMRs) of acute pancreatitis and the average annual percentage changes (AAPCs) among US adults from 1999 to 2020.

We obtained data from the Multiple Cause of Death database on the Centers for Disease Control and Prevention Wide-ranging Online Data for Epidemiologic Research,^3^ which provides mortality data from all death certificates filed in the 50 states and the District of Columbia. Each death certificate contains a single underlying cause of death and up to 20 additional multiple causes. Deaths related to acute pancreatitis as a contributing cause were identified using the international classification of diseases-10 codes K85.x. Mortality rates were age-adjusted to the 2000 US Standard Population and reported as deaths per 100,000 population. Our study aimed to examine mortality rates among US adults. We focused on populations aged 25–34 years (rather than 15–24 years) and older since age-adjusted rates are available for 10-year age groups.

Demographic characteristics, including age, sex, race and ethnicity, and rurality, were obtained from death certificates. Race and ethnicity were classified into the following categories: non-Hispanic White, non-Hispanic Black (NHB), (non-Hispanic) Asian American and Pacific Islander, (non-Hispanic) American Indian and Alaska Native (AI/AN), and Hispanic. Rurality was classified based on the 2013 National Center for Health Statistics Urban-Rural Classification Scheme: large metropolitan (≥1 million population), small- or medium-sized metropolitan (50,000–999,999 population), and rural areas (<50,000 population).

We presented AAMRs of acute pancreatitis and analyzed these rates across various demographic characteristics. AAPCs were calculated using the Joinpoint Regression Program version 5.0.2. Graphs were created using R version 4.3.2.

Between 1999 and 2020, there were 126,360 deaths related to acute pancreatitis among 4,473,854,489 US adults (AAMR, 2.70 deaths per 100,000 population; 95% confidence interval (CI), 2.69–2.72). Mortality rates were highest among older adults (≥65 years) (AAMR, 7.86; 95% CI, 7.80–7.92), males (AAMR, 3.35; 95% CI, 3.33–3.38), AI/ANs (AAMR, 3.98; 95% CI, 3.74–4.21), NHBs (AAMR, 3.48; 95% CI, 3.42–3.53), and those living in rural areas (AAMR, 3.26; 95% CI, 3.22–3.30) (Figure). Estimates are available in Table A1. AAMRs among US adults declined from 3.31 (95% CI, 3.22–3.39) in 1999 to 2.25 (95% CI, 2.19–2.31) in 2019 (AAPC, −1.5%; 95% CI, −2.2% to −1.0%), although there was a notable increase to 2.72 (95% CI, 2.69–2.72) in 2020 (20.9% increase). All demographic groups, except for younger adults and AI/ANs, exhibited a similar trend.

**Figure:**
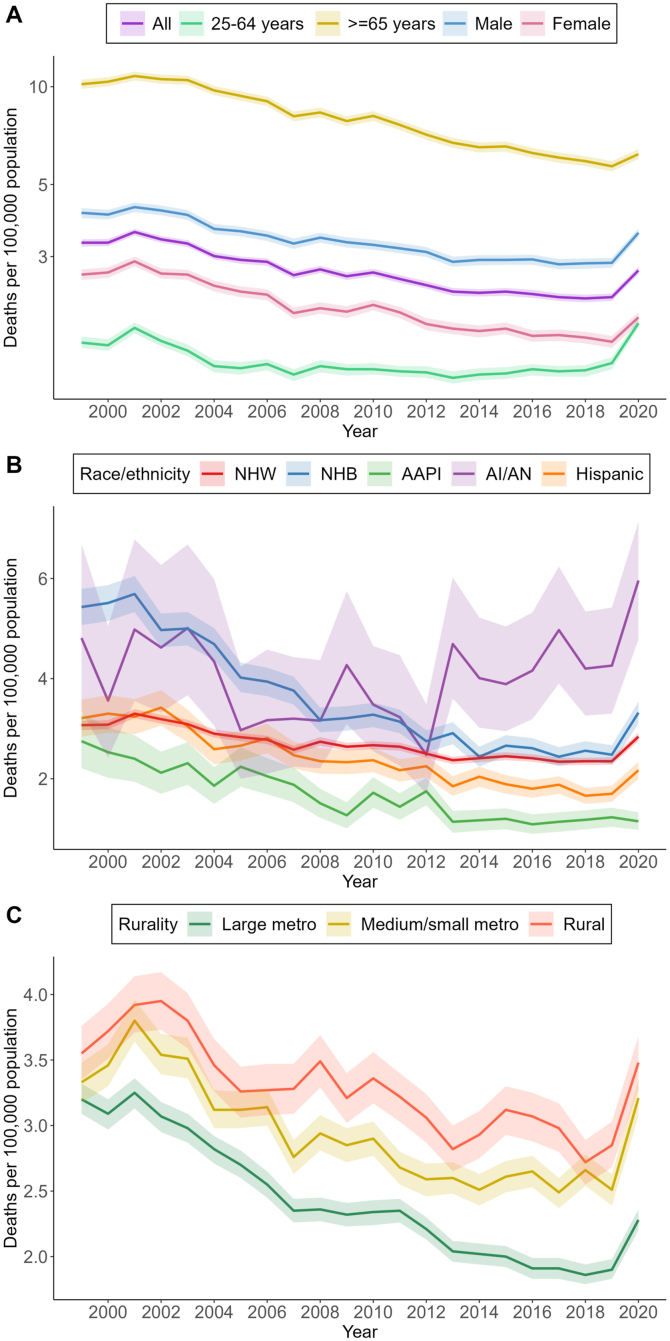
Trends in acute pancreatitis-related mortality among US adults by demographic characteristics, 1999–2020.

Most demographic groups experienced increases in mortality rates from 2019 to 2020. The most pronounced increases were observed among younger adults (1.41 to 1.87; 32.6% increase), NHBs (2.48 to 3.32; 33.9% increase), and AI/ANs (4.26 to 5.96; 39.9% increase). In addition, AAMRs for medium/small metropolitan populations rose from 2.51 to 3.21 (27.9% increase) and for rural populations from 2.85 to 3.48 (22.1% increase).

From 1999 to 2020, AAMRs for AI/ANs showed an overall nonsignificant increase (AAPC, 0.6%; 95% CI, −2.7%–6.3%), whereas other racial/ethnic groups had significant declines. NHBs (AAPC, −2.9%; 95% CI, −5.1% to −1.7%) and Asian American and Pacific Islanders (AAPC, −4.2%; 95% CI, −6.2% to −2.4%) experienced the greatest decline in AAMRs. Large metropolitan population saw a downtrend in AAMRs throughout the study period (AAPC, −1.8%; 95% CI, −3.1% to −1.3%). In contrast, medium/small metropolitan (AAPC, −0.8%; 95% CI, −2.3%–0.1%) and rural populations (AAPC, −0.6%; 95% CI, −2.9%–1.5%) were noted to have overall nonsignificant decreases in AAMRs.

Among US adults, acute pancreatitis-related mortality has declined over the past 2 decades, thanks to improved detection of severe cases, advances in therapeutic measures, and better supportive and intensive care. Nonetheless, disparities in mortality persist across demographic characteristics. Our analysis revealed that the mortality burden is substantially higher in racial and ethnic minorities, particularly NHBs and AI/ANs, as well as rural populations. This disparity can be partly explained by differences in exposure to risk factors and access to care. Reports suggest that the rate of excessive alcohol consumption is higher among NHBs and AI/ANs,^4^ which may be influenced by cultural and social norms, easier access to alcoholic beverages, and inadequate social support. Additionally, the rate of gallstone disease is higher in AI/ANs,^5^ which may lead to a disproportionately high incidence of gallstone pancreatitis. Lower health literacy and limited access to health-care providers likely contribute to higher mortality rates in rural populations.^6^

Mortality disparities were further exacerbated by the Coronavirus disease 2019 (COVID-19) pandemic, as NHBs, AI/ANs, and rural populations experienced greater increases in acute pancreatitis-related mortality compared to other groups. Social isolation, psychological and financial strains, and disrupted routines during the pandemic have been shown to lead to increased alcohol consumption in these populations.^7^ Additionally, the risk of COVID-19 was higher among racial and ethnic minorities, who often could not practice social distancing due to their roles as essential workers and residence in densely populated areas.^8^ Concomitant COVID-19 was associated with higher acute pancreatitis-related mortality,^9^ further worsening disparities.

This study has several limitations. First, there is potential for misclassification in death certificate documentation. Second, we were unable to assess different etiologies of acute pancreatitis due to the small sample size. Third, the 2000 US standard population may not reflect the current age distribution. Nevertheless, this age-adjustment method remains statistically robust and has been widely accepted since its introduction in 1999.

In conclusion, while acute pancreatitis-related mortality rates among US adults have declined, significant disparities persist and were exacerbated by the COVID-19 pandemic. These trends underscore the urgent need for targeted public health interventions to aid vulnerable populations.

## Supplementary materials

Table A1
