# Supplementary material for: Trends in Acute Pancreatitis-Related Mortality Among US Adults from 1999 to 2020
Source: Gastro Hep Adv. 2025 Jan 10;4(5):100615. doi: 10.1016/j.gastha.2025.100615 (PMC12008571; doi:10.1016/j.gastha.2025.100615)
Supplement: Table A1 [file mmc1.docx]

| **Table A1. Acute pancreatitis-related mortality among US adults by demographic characteristics, 1999-2020** | | | | | | | |
| --- | --- | --- | --- | --- | --- | --- | --- |
|  | **AAMR (95% CI)** | | | | **AAPC (95% CI)** | | **% change** |
|  | 1999 | 2019 | 2020 | 1999-2020 | 1999-2019 | 1999-2020 | 2019-2020 |
| **All** | 3.31 (3.22-3.39) | 2.25 (2.19-2.31) | 2.72 (2.65-2.79) | 2.70 (2.69-2.72) | -1.5 (-2.1 to -1.0) | -1.0 (-1.6 to -0.4) | 20.9 |
| **Age** |  |  |  |  |  |  |  |
| 25-64 years | 1.63 (1.57-1.70) | 1.41 (1.35-1.47) | 1.87  (1.81-1.94) | 1.45  (1.44-1.47) | -0.2 (-0.8 to 0.4) | 0.7 (-0.1 to 1.3) | 32.6 |
| ≥65 years | 10.18 (9.85-10.52) | 5.69 (5.49-5.90) | 6.20  (5.99-6.42) | 7.86  (7.80-7.92) | -2.6 (-3.2 to -2.1) | -2.4 (-3.1 to -1.8) | 9.0 |
| **Sex** |  |  |  |  |  |  |  |
| Male | 4.09 (3.94-4.23) | 2.87 (2.77-2.97) | 3.55 (3.44-3.66) | 3.35 (3.33-3.38) | -1.6 (-2.4 to -1.0) | -1.0 (-2.3 to -0.3) | 23.7 |
| Female | 2.64 (2.54-2.74) | 1.64 (1.57-1.71) | 1.95 (1.87-2.02) | 2.09 (2.08-2.11) | -2.4 (-3.4 to -1.4) | -1.8 (-3.3 to -1.1) | 18.9 |
| **Race/ethnicity** |  |  |  |  |  |  |  |
| NHW | 3.07 (2.97-3.16) | 2.35 (2.28-2.43) | 2.84 (2.75-2.92) | 2.68 (2.67-2.70) | -1.2 (-1.9 to -0.7) | -0.7 (-1.9 to -0.2) | 20.9 |
| NHB | 5.43 (5.07-5.79) | 2.48 (2.29-2.67) | 3.32 (3.10-3.54) | 3.48 (3.42-3.53) | -3.5 (-5.2 to -2.3) | -2.9 (-5.1 to -1.7) | 33.9 |
| AAPI | 2.75 (2.21-3.30) | 1.23 (1.04-1.42) | 1.15 (0.98-1.33) | 1.49 (1.44-1.55) | -4.2 (-6.2 to -2.4) | -4.2 (-6.2 to -2.4) | -6.5 |
| AI/AN | 4.81 (3.35-6.69) | 4.26 (3.30-5.42) | 5.96 (4.78-7.13) | 3.98 (3.74-4.21) | 0.4 (-3.0 to 6.5) | 0.6 (-2.7 to 6.3) | 39.9 |
| Hispanic | 3.21 (2.84-3.58) | 1.70 (1.54-1.86) | 2.17 (2.00-2.34) | 2.26 (2.21-2.31) | -3.0 (-4.3 to -1.1) | -2.4 (-4.2 to -1.2) | 27.6 |
| **Rurality^a^** |  |  |  |  |  |  |  |
| Large metropolitan areas | 3.20 (3.09-3.32) | 1.90 (1.83-1.98) | 2.28 (2.20-2.36) | 2.38 (2.36-2.40) | -2.4 (-3.1 to -1.8) | -1.8 (-3.1 to -1.3) | 20.0 |
| Medium/small metropolitan areas | 3.33 (3.17-3.48) | 2.51 (2.39-2.63) | 3.21 (3.07-3.34) | 2.90 (2.88-2.93) | -1.2 (-2.3 to -0.2) | -0.8 (-2.3 to 0.1) | 27.9 |
| Rural areas | 3.55 (3.35-3.76) | 2.85 (2.68-3.03) | 3.48 (3.28-3.68) | 3.26 (3.22-3.30) | -1.1 (-2.8 to 1.6) | -0.6 (-2.9 to 1.5) | 22.1 |
| Abbreviations: AAMR, age-adjusted mortality rate; AAPC, average annual percentage change; AAPI, Asian American and Pacific Islander; AI/AN, American Indian and Alaska Native; CI, confidence interval; NHB, non-Hispanic Black; NHW, non-Hispanic White.  ^a^ Rurality was categorized based on the 2013 National Center for Health Statistics (NCHS) Urban-Rural Scheme. Large metropolitan areas were defined as population ≥1 million; medium/small metropolitan areas were defined as population 50,000-999,999; rural areas were defined as population <50,000. | | | | | | | |
